# Surgical oncological emergencies in octogenarian patients

**DOI:** 10.3389/fonc.2023.1268190

**Published:** 2023-11-29

**Authors:** Alberto Friziero, Cosimo Sperti, Federica Riccio, Irene Sole Zuin, Lorenzo Vallese, Simone Serafini, Alessandra Amico, Valeria Valli, Chiara Da Re, Nicola Baldan, Michele Valmasoni, Gianfranco Da Dalt

**Affiliations:** ^1^ Department of Surgery, Oncology and Gastroenterology, 1^st^ Surgical Clinic, University of Padua, Padua, Italy; ^2^ Department of Surgery, Oncology and Gastroenterology, 2nd Surgical Clinic, University of Padua, Padua, Italy

**Keywords:** age, colectomy, colon cancer, emergency, octogenarians, surgical oncology

## Abstract

**Background:**

Surgical oncological emergencies represent a frequent challenge in acute settings, with postoperative courses characterized by high morbidity and mortality. An accurate selection of patients who could benefit from surgery is essential to avoid unnecessary invasive treatment. In this study, we tried to determine if advanced age (>80 years) represents a risk factor for negative short-term outcome in patients undergoing emergency surgery for acute abdominal oncological illness.

**Methods:**

We retrospectively analyzed the records of patients who underwent emergency oncological surgery at the Department of Acute Care Surgery of Padua General Hospital from January 2018 to December 2022. One hundred two cancer patients were included in the study. Among them, 42 were aged ≥80 years (41%). Multiple preoperative and postoperative parameters were recorded, and the follow-up period was at least 90 days. Multivariate logistic regression analyses were used to identify factors associated with short-term postoperative outcomes.

**Results:**

In the octogenarian group, 30-day mortality was 11% vs. 9.5% in the younger group [p = not significant (*ns*)] and 90-day mortality was 17.6% in the octogenarian group vs. 20.5% in the younger group (p = *ns*). Postoperative morbidity and hospital length of stay were not significantly different in the two groups. Low albumin levels [odds ratio (OR) 30.6, 9.51–87.07] and elevated lactate dehydrogenase (LDH) levels (OR 26.4, 9.18–75.83) were predictive for short-term mortality in surgical oncological emergencies.

**Conclusion:**

Advanced age is not a risk factor for negative outcomes in surgical oncological emergencies. Therefore, surgical options should be considered in octogenarians with oncological emergencies and acceptable clinical conditions. Serum albumin levels and LDH can help predict the postoperative outcome after surgery for oncological emergencies.

## Introduction

An oncologic emergency may be defined as an acute life-threatening condition that has developed directly or indirectly as a result of cancer or cancer treatment (1). Perforation, occlusion, and gastrointestinal bleeding are the most common abdominal surgical emergencies in cancer patients (2). Decisions regarding treatment in emergencies are often not easy to make. In the absence of specific guidelines, the optimal method for objective evaluation and decision-making would be discussed in a multidisciplinary cancer conference. This makes surgical oncological emergency one of the most critical scenarios for acute care surgeons (3). It is essential to define the prognosis of both emergency and the cancer stage and to consider the patient’s performance score when deciding on the treatment.

The octogenarian population is expanding in industrialized countries, and at this point, the acute care surgery segment is growing. Emergency general surgery operations in elderly patients are overall associated with significant morbidity and mortality owing to underlying frailty, comorbidities, and reduced physiological reserve. It is known that older patients, in particular octogenarians, have poorer outcomes, with up to 44% mortality reported (4, 5).

In contrast, the only few studies focused on surgical oncological emergencies did not report chronological age as a predictive factor for negative surgical outcomes (6, 7).

The purpose of this retrospective study was first to investigate the outcome of octogenarian patients who underwent emergency operations for oncological acute illnesses in our institution and second to identify preoperative patient-related factors predicting postoperative mortality. As the geriatric population increases, we think that it is important to analyze if this parameter has a relevant influence on postoperative outcomes in older surgical patients presenting with acute malignancy-related symptoms.

## Patients and methods

**Figure 1:**
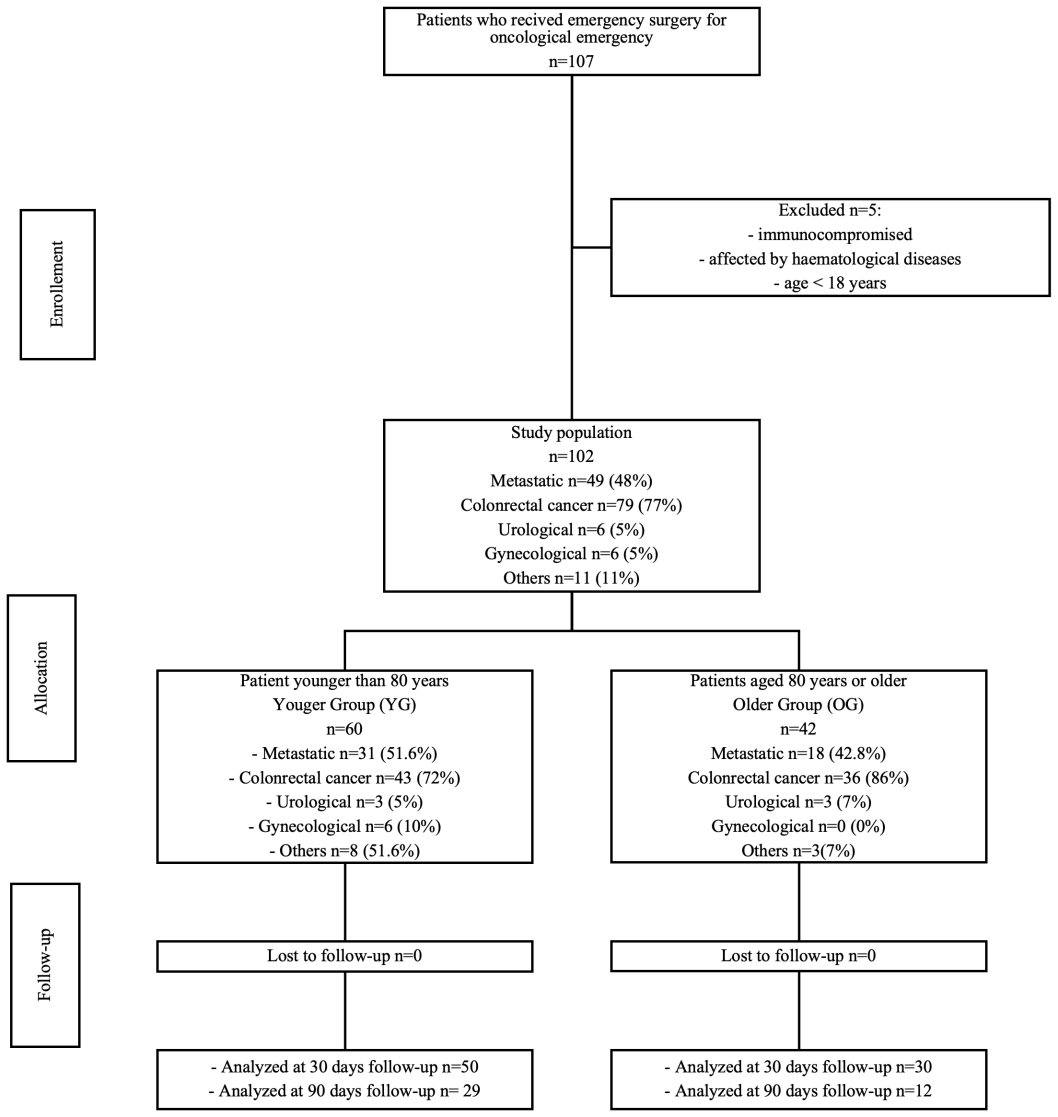
Distribution of the population.

Between January 2018 and December 2022, 102 patients underwent emergent surgery at our institution for abdominal oncologic emergencies. Patients were divided into two groups: the octogenarian group (OG) (n = 42), consisting of patients aged 80 or older, and the younger group (YG) (n = 60), consisting of patients under 80 years of age (**Figure 1**).

Inclusion criteria for the study were as follows: (i) age ≥18 years old; surgery for abdominal oncologic emergency for (ii) symptoms caused by any type of solid malignant disease (including primary onset) or for (iii) symptoms caused by current or previous cancer treatment (previous surgery, radiation therapy, chemotherapy, or drug-targeted therapy). Patients requiring emergency surgery for causes that could not be related directly to malignant disease or cancer treatment were excluded from the analysis (i.e., adherences or incisional hernias due to previous surgery). Finally, immunocompromised or hematological patients were excluded.

The two groups were compared in terms of (i) demographic characteristics, (ii) preexisting comorbidity, (iii) patient’s performance and functional status, (iv) cancer history, (v) preoperative laboratory tests, (vi) surgical procedure, and (vii) postoperative outcomes.

Comorbidity status before the emergency consultation was determined by Charlson Comorbidity Index (CCI) (8, 9). Performance status was assessed by the Eastern Cooperative Oncology Group performance score (ECOG-PS) (10), and functional status was determined by the American Society of Anesthesiologists (ASA) and divided in different physical status classes 1, 2, 3, or 4 (11). Cancer histology, chemotherapy treatments before surgery, and last oncological status based on RECIST criteria (12) were recorded for each patient.

The following biochemical lab tests and inflammatory markers were analyzed: serum leukocyte count, C-reactive protein (CRP), hemoglobin, creatinine, albumin, and lactate dehydrogenase (LDH). Systemic inflammatory response syndrome (SIRS) criteria were obtained (13, 14).

The indications for surgery were classified into the three most common surgical oncological emergencies (obstruction, visceral perforation, and gastrointestinal bleeding) (6). The intent of surgical treatment was differentiated into curative resection, defined as a tumor’s resection with pathologically confirmed negative margins and no macroscopic tumor residues, or palliative when a complete oncological resection has not been achieved.

The standardized Clavien–Dindo classification of surgical complications (15) is applied as a single and widely used tool to assess and report postoperative morbidity, and 90-day mortality was analyzed for each patient. Median follow-up was 4 months, ranging from 0 to 49 months.

### Statistical analysis

Clinicopathological characteristics were expressed as medians with interquartile ranges (IQRs) as appropriate and compared using chi-square or Fisher exact test for categorical variables; the Mann–Whitney test was used for continuous variables. Cutoff scores for ECOG (10), ASA (11), and CCI (8) were obtained from previous established experiences, whereas for biochemical variables, they were calculated from the receiver operating characteristic (ROC) curve analysis. The optimal cutoff was identified as the point of intersection nearest the top left-hand corner between the ROC curve and a diagonal line drawn from the top right-hand corner to the bottom left-hand corner of the graph. Survival curves were constructed with the Kaplan–Meier method, and differences in survival curves were compared by log-rank test. Multivariate logistic regression analysis was performed to identify factors associated with 90-day mortality. This model was constructed by incorporating all of the preoperative variables that were statistically significant in the univariate analysis.

All data were processed using GraphPad Prism 9 (GraphPad Software Inc., CA, USA), and a p < 0.05 was considered significant.

This study was approved by the local institutional review board and followed local protocols in accordance with the Declaration of Helsinki.

## Results

During the study period, 102 patients were included: 47 were men (46%) and 55 were women (54%). Women were the most predominant gender in the octogenarian group (76.2%). Nearly 41% (n = 42) of the patients were aged 80 or older: 33 patients were between the ages of 80 and 89 years, and nine patients were aged 90 or older. The oldest patient was 98 years old, and the median age of the whole cohort was 75 (range 39–98).

The clinicopathological and operative features of the study population and a comparison between octogenarian and younger groups are reported in **Tables 1**, **2**.

**Table 1:** Baseline and clinicopathological characteristics of the study population.

		Entire Cohort, n=102	Octogenarian Group, n=42	Younger Group, n=60	P
Sex (M)		47 (46%)	10 (23.8%)	37 (61.6%)	0.001
ECOG (median, IQR)		1 (0-1)	1 (1-1)	0 (0-3)	0.02
CCI (median, IQR)		6 (4-9)	5 (4-9)	6 (2-9)	0.38
ASA (median, IQR)		3 (2-3)	3 (3-3)	2.5 (2-3)	0.002
SIRS		22 (21.6%)	10 (23.8%)	12 (20%)	0.8
Leukocytes (median, IQR)		10 (6-13)	11 (9-14)	9.5 (5.2-12)	0.11
Hemoglobin (median, IQR)		11.5 (10-13)	11 (9-12)	12 (10-13)	0.007
Creatinine (median, IQR)		80 (69-107)	90 (70-125)	78 (68-93)	0.07
LDH (median, IQR)		200 (154.3-250)	206.5 (178-230)	200 (150-290)	0.9
CRP (median, IQR)		23 (5-68)	25 (4-55)	23 (6-96)	0.49
Albumin (median, IQR)		30 (21.5-35)	27 (21-30)	30 (23-36)	0.1
New diagnosis		70 (69%)	33 (78%)	37 (62%)	0.23
Past diagnosis		32 (31%)	9 (12%)	23 (38%)	
	Stable disease	7 (22%)	3 (33%)	4 (17%)	
	Progression disease	25 (78%)	6 (67%)	19 (83%)	
Isotype					
	Colorectal	79 (77%)	36 (86%)	43 (72%)	0.14
	Urological	6 (5%)	3 (7%)	3 (5%)	
	Gynecological	6 (5%)	0	6 (10%)	
	Others	11 (13%)	3 (7%)	8 (15%)	
Metastatic cancer (Stage IV)		49 (48%)	18 (42.8%)	31 (51.6%)	0.42
Past chemotherapy treatment		27 (26%)	4 (9,5%)	23 (38%)	0.001
Active chemotherapy		17 (16.6%)	2 (4.8%)	15 (25%)	0.007
Antiangiogenic treatment		9 (8.7%)	2 (4.8%)	7 (11.6%)	0.23

EGOG, Eastern Cooperative Oncology Group; CCI, Charlson Comorbidity Index; ASA, American Society of Anesthesiologists; SIRS, systemic inflammatory response syndrome; LDH, lactate dehydrogenase; CRP, C-reactive protein.

**Table 2:** Operative variables, postoperative variables, and survival analysis.

		Entire Cohort, n=102	Octogenarian Group, n=42	Younger Group, n=60	p
Indication for surgery					0.14
	Obstructions	77 (70.5%)	30 (71.5%)	42 (70%)	
	Perforations	22 (21.1%)	8 (19%)	14 (23%)	
	Bleedings	8 (7.8%)	4 (9.5%)	4 (7%)	
Type of surgery					0.81
	Palliative	52 (51%)	20 (47.6%)	30 (50%)	
	Resective	50 (49%)	22 (52.4%)	30 (50%)	
Laparoscopy		31 (30%)	8 (19%)	23 (38%)	0.03
Ostomy		50 (49%)	19 (45%)	31 (51.6%)	0.89
Clavien–Dindo (median, IQR)		0 (0-2)	0 (0-3)	0 (0-2)	0.50
Overall morbidity		45 (44%)	17 (40%)	28 (46%)	0.68
Major morbidity		24 (23.5%)	8 (19%)	16 (26%)	0.09
Length of stay (days) (median, IQR)		9 (6-12.5)	9 (6-13)	9 (6-12)	0.95
Survival analysis					
Overall survival (months) (median, IQR)		17 (6.8-25)	19 (7.6-28.5)	16 (6.5-24)	0.49
30-day mortality		22 (21.5%)	12 (11%)	10 (9.5%)	0.18
90-day mortality		39 (38%)	18 (17.6%)	21 (20.5%)	0.10

The most prominent type of cancer was colorectal carcinoma (77%), followed by urological (5%) and gynecological cancer (5%). Seventy patients (69%) received their first diagnosis of cancer during the first onset, and 49 (48%) had metastatic disease (Stage IV according to TNM classification) (16). Seventeen patients (16.6%) were on systemic chemotherapy at admission. Obstruction was the most frequent surgical oncological emergency, with no difference between the two groups.

The median ECOG-PS in the octogenarian group was 1 (IQR 1–1) compared with 0 (IQR 0–3) in the younger group, and this difference was statistically significant (p < 0.02). By comparing the ASA functional status in the two groups, the elderly group presented a significantly higher number of patients with ASA class 3 (IQR 3–3) compared to that in the younger group (p = 0.002). In contrast, there was no statistically significant difference concerning the CCI.

By analyzing clinical and laboratory data upon first presentation, we found that approximately 20% of all patients met SIRS criteria, with no significant differences between elderly and non-elderly patients.

Indications for surgery, type of operation, and outcome are reported in **Tables 2**, **3**. The most frequently performed procedure was left hemicolectomy (30%), and laparoscopy was performed in only 31 patients, most of them in the younger group (p = 0.03). Curative resections were achieved in approximately half of the patients, with no statistically significant difference between octogenarian and younger patients.

**Table 3:** Surgical operations performed during the study period.

	Entire Cohort, n=102
Intestinal bypass	3 (2.9%)
Small bowel resection	7 (6.8%)
Loop ileostomy	16 (15.7%)
Loop colostomy	12 (11.7%)
Right hemicolectomy	25 (24.5%)
Left hemicolectomy	31 (30%)
Rectal anterior resection	2 (1.9%)
Partial gastrectomy	2 (1.9%)
Explorative laparotomy	4 (3.9%)

The overall morbidity was the same in the two groups (40% vs. 46%), mostly represented by postoperative ileus and wound infections. The most frequent major morbidity (Clavien–Dindo ≥3b) was anastomotic leak, with a similar rate in the two groups (19% vs. 26%). Even if not statistically significant (p=0.09), it is to underline that the complication and major morbidity rates were higher in the younger group.

**Figure 2:**
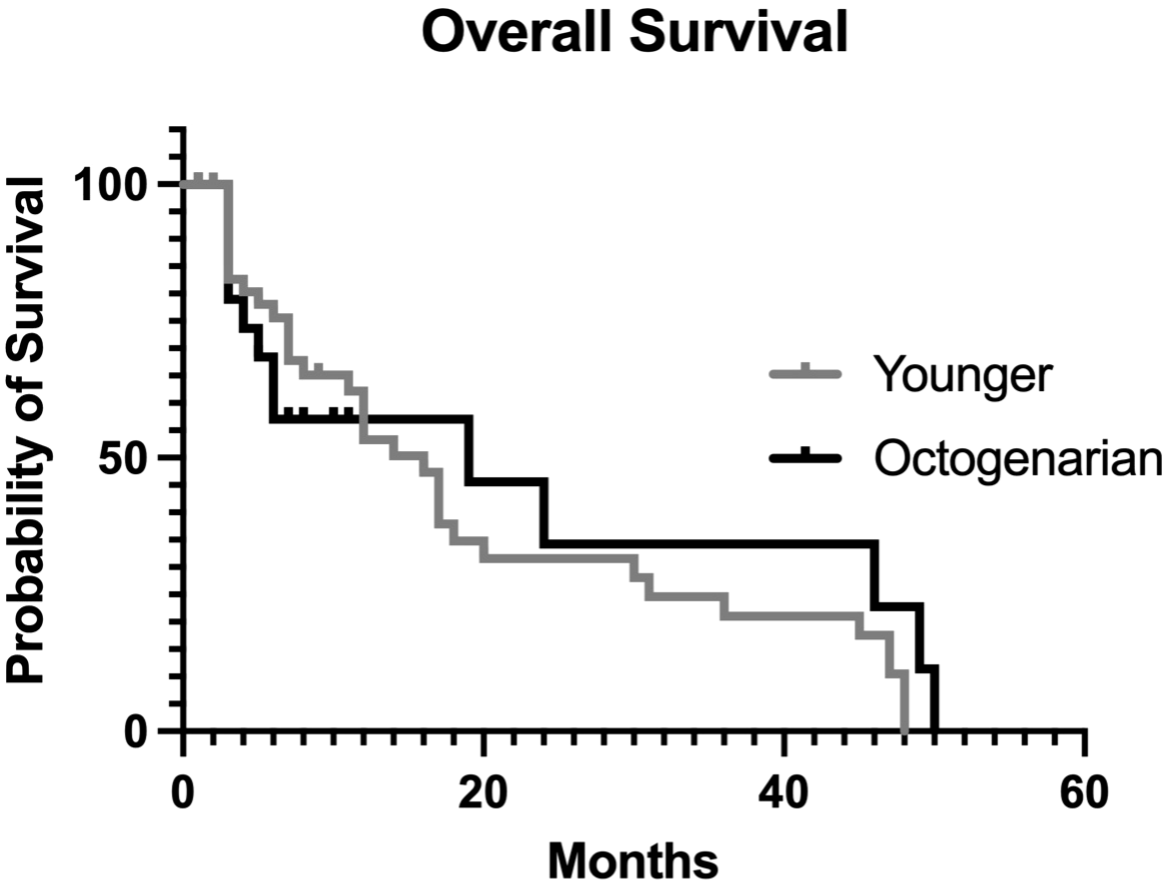
Overall actuarial survival curves comparing patients under 80 years to patients aged >80 years.

Median overall survival was 17 (IQR 6.8–25) months, with 30-day mortality and 90-day mortality rates of 21% and 38%, respectively. There were no statistically significant differences between the two groups (**Figure 2**).

From our analysis, the most prominent cause of death was cancer progression, followed by postoperative surgical complications. Death due to medical complications (cardiovascular, pulmonary, renal, or infectious) associated with 30- and 90-day mortality was lower than 10% of the total, without any statistical difference between the younger and the octogenarian group (**Table 4**).

**Table 4:** Causes of death.

		Entire Cohort, n=102	Octogenarian Group, n=42	Younger Group, n=60	P
30-day mortality		22 (21.5%)	12 (11%)	10 (9.5%)	
	Surgical complication	6 (27%)	3 (25%)	3 (30%)	0.68
	Cancer progression	13 (59%)	7 (59%)	6 (60%)	0.32
	Cardiological complication	1 (4.5%)	1 (8%)	0 (0%)	0.41
	Pulmonary complication	1(4.5%)	0 (0%)	1 (10%)	0.9
	Renal complication	1 (4.5%)	1 (8%)	0 (0%)	0.41
	Sepsis	0 (0%)	0 (0%)	0 (0%)	0.9
90-day mortality		39 (38%)	18 (17.6%)	21 (20.5%)	
	Surgical complication	4 (10.3%)	1 (5.6%)	3 (14.3%)	0.64
	Cancer progression	28 (71.7%)	13 (72.2%)	15 (71.3%)	0.50
	Cardiological complication	3 (7.7%)	2 (11.1%)	1 (4.8%)	0.56
	Pulmonary complication	1 (2.6%)	0 (0%)	1 (4.8%)	0.9
	Renal complication	2 (5.1%)	2 (11.1%)	0 (0%)	0.16
	Sepsis*	1 (2.6%)	0 (0%)	1 (4.8%)	0.9

* the cause of death was sepsis due to a urinary tract infection.

**Figure 3:**
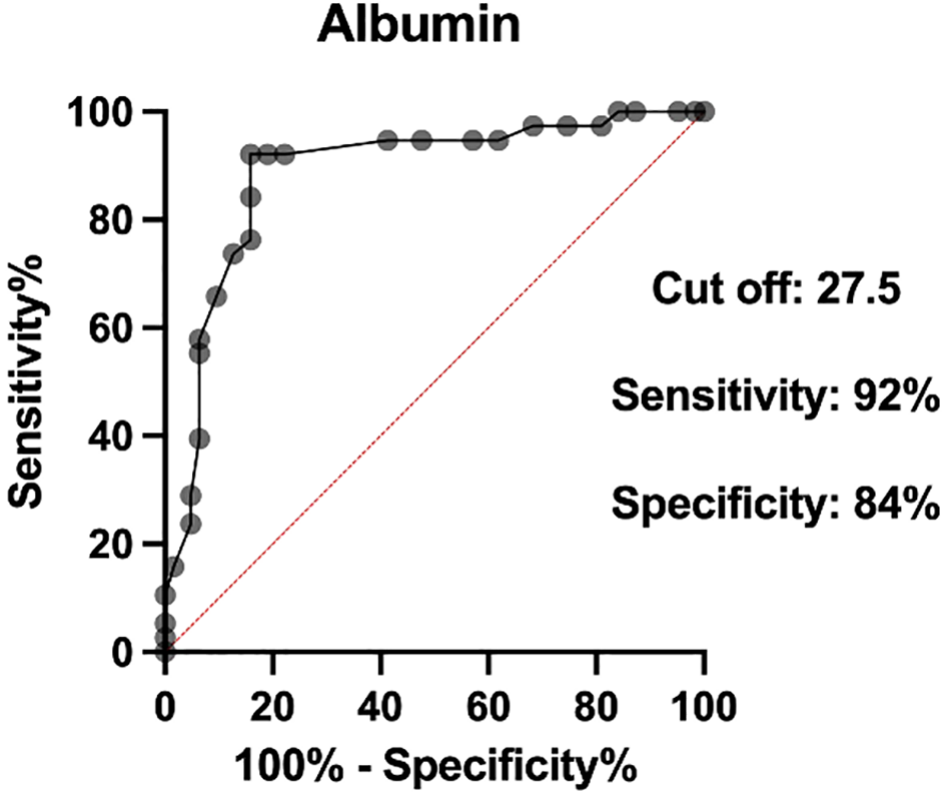
Receiver operator characteristic (ROC) curves of serum albumin levels.

**Figure 4:**
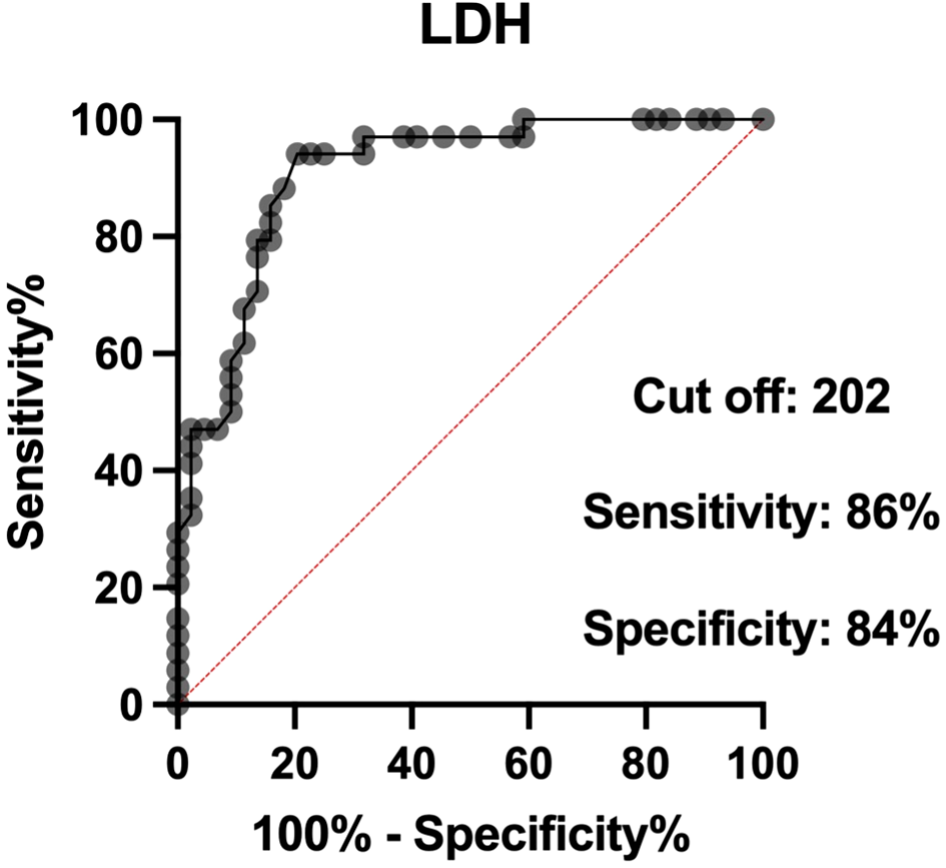
Receiver operator characteristic (ROC) curves of serum LDH levels.

Two biomarkers were examined, and ROC curve analysis was set to identify the optimal cutoff values for albumin and LDH. The optimal cutoff values were as follows: 27.5 for albumin (AUC 0.88, sensitivity 92%, specificity 84%) and 202 for LDH (AUC 0.90, sensitivity 86%, specificity 84%) (**Figures 3**, **4**).

The univariate and multivariate analyses of 90-day mortality are shown in **Table 5**. There was a statistically significant association between mortality and ECOG-PS >0 (p = 0.0001), CCI >6 (p = 0.008), creatinine level >78 µmol/L (p = 0.005), albumin <27.5 g/L (p < 0.0001), LDH >202 U/L (p < 0.0001), CRP >23.5 mg/L (p = 0.0002), presence of SIRS syndrome (p = 0.0003), cancer progression (p < 0.0001), metastatic disease (p = 0.0002), and past chemotherapy treatment (p = 0.002). All of these variables were included in the multivariate analysis. Albumin level <27.5 g/L and raised LDH >202 U/L remained as independent predictors of 90-day mortality.

**Table 5:** Univariate and multivariate analyses of preoperative factors associated with 90-day mortality.

	90-day mortality	OR (95% CI)	p	OR (95% CI)	p
Age >80	18 (17.65%)	1.84 (0.83-4.13)	0.14		
Sex (M)	15 (14.7%)	1.65 (0.71-3.71)	0.3		
ECOG >0	37 (36.2%)	19.2 (4.42-84.17)	0.0001	28.1 (0.9-65.68)	0.09
CCI >6	23 (22.5%)	3.09 (1.30-7.02)	0.008	2.7 (0.1-106.1)	0.69
ASA >3	6 (5.8%)	5.54 (1.2-27.7)	0.051		
Leukocytes × 11.7 × 10^9^/L	19 (18.6%)	1.26 (0.58-2.70)	0.68		
Hemoglobin <11.5 g/L	18 (17.6%)	1.28 (0.55-2.75)	0.63		
Creatinine >78 µmol/L	26 (25.5%)	3.25 (1.36-7.13)	0.005	1 (0.96-1.02)	0.66
Albumin <27.5 g/L	35 (34.3%)	30.6 (9.51-87.07)	< 0.0001	4.5 (1.32-9.5)	0.02
LDH >202 U/L	32 (32%)	26.4 (9.18-75.82)	< 0.0001	6 (2.5-12.54)	0.009
CRP >23.5	28 (27.45%)	5.09 (2.03-12.18)	0.0002	1.02 (0.06-13-12)	0.97
SIRS	16 (15.7%)	6.6 (2.34-19.21)	0.0003	0.01 (0.005-3.68)	0.07
Metastatic cancer (Stage IV)	28 (27.45%)	5.09 (2.03-12.18)	0.0002	14.7 (0.8-66.8)	0.09
Progressive disease	20 (19.8%)	12 (4.05-31.55)	< 0.0001	4.9 (0.08-5.80)	0.45
Past chemotherapy treatment	17 (16.67%)	4.09 (1.57-9.84)	0.002	0.41 (0.03-29.9)	0.68
Active chemotherapy	9 (8.8%)	2.05 (0.72-6.18)	0.18		

EGOG, Eastern Cooperative Oncology Group; CCI, Charlson Comorbidity Index; ASA, American Society of Anesthesiologists; LDH, lactate dehydrogenase; CRP, C-reactive protein; SIRS, systemic inflammatory response syndrome.

## Discussion

The present study aimed to evaluate the outcome after surgery for oncological emergencies in 102 patients, 42 of whom (41%) were octogenarians. Despite the octogenarians presenting more comorbidities and worse performance status, which were consistent with significantly higher ASA scores and lower ECOG-PS, no significant differences were observed in terms of survival and postoperative complications. These results suggest that the prognosis of surgical oncological emergencies is probably determined by other factors as compared to other non-oncological emergencies where the physiological status plays a key role in postoperative outcomes. A proof of this concept was derived from the results of our multivariate analysis where high LDH and low albumin levels were the only predictors of short-term survival.

The main goal of surgeons when dealing with acute oncological illness is to offer the most appropriate treatment with clinical benefit and the improvement of the residual quality of life. For this, an appropriate patient selection is mandatory. Octogenarians are a particular interest group; they are frequently frail or with multiple comorbidities (17). Also, they are exposed to a higher rate of complications when compared to younger patients (18, 19). In a systematic review and meta-analysis of Kennedy et al. (20), there is strong evidence to suggest that frailty in the older population predicts postoperative mortality, morbidity, prolonged length of hospital stay, and the loss of independence.

In the report by St-Louis et al. (9), focused on emergency surgery, chronological age together with CCI, ECOG-PS, and ASA score impacted the postoperative outcome in terms of a higher rate of postoperative morbidity and mortality, but with the limit of not distinguishing between oncological and non-oncological causes. In another series, age alone is recognized as an independent risk factor for morbidity and mortality in acute care general surgery (21).

In our experience, classical markers of poor prognosis, such as metastatic disease and cancer progression, appear associated with 90-day mortality in univariate analysis, but they do not reach statistical significance as independent predictive factors. This is probably related to an adequate preoperative patient selection and an appropriate surgical procedure. Indeed, approximately half of the patients received palliative treatment finalized to solve the acute problem, avoiding overtreatment.

Similar results concern typical markers of acute inflammatory response, notoriously correlated with poor prognosis in acute care surgery (22), as CRP and SIRS, that were significantly associated with 90-day mortality in univariate but not in multivariate analysis. High serum LDH and low albumin levels are historically considered as negative predictors in cancer patients, first for their role as biomarkers of cancer metabolism and second for their relationship with cancer cachexia. However, this concept has been challenged in the last decade, redefining their role as inflammation biomarkers in cancer patients independently of malnutrition or cancer metabolism (23). Low serum albumin seems to be directly related to elevated levels of circulating pro-inflammatory cytokines (as interleukin 6) that inhibit hepatocyte synthesis (24). Moreover, inflammation increases capillary permeability, promoting the escape of serum albumin, leading to an indirect decrease of serum levels. In a Taiwanese study of 3,800 colon cancer patients, hypoalbuminemia predicted a higher rate of 30-day mortality and was associated with more common wound healing and anastomotic complications, as well as pulmonary and urinary morbidities (24). LDH tends to be positively correlated with the systemic inflammatory response in various solid malignancies (25). LDH levels increase with tumor hypoxia and necrosis and are positively correlated with abnormal activation of hypoxia-inducible factor-1 (HIF-1), an oxygen-sensitive factor, upregulated in low-oxygen tumor microenvironments. HIF-1 has been shown to exacerbate certain sepsis infection models through induction of pro-inflammatory cytokines and chemokines (26).

This evidence could explain the great relevance of LDH and albumin levels in oncological emergencies as biomarkers of cancer progression and simultaneously systemic inflammatory response. For this, the measurement of LDH and albumin levels in emergency settings could serve as objective prognostic parameters to assess the clinical outcome and define the most appropriate surgical treatment. The importance to check albumin and LDH levels as objective prognostic parameters in the emergency setting has been previously emphasized (6): in a series of 207 patients presenting surgical oncologic emergencies, raised LDH (p < 0.001) and low albumin levels (p = 0.002) were associated with 30-day and 90-day mortality, respectively.

The retrospective nature and the relatively small population sample are the main limitations of this study. Furthermore, the heterogeneous characteristics of oncological presentations do not allow to create any solid prediction model. However, we believe that this study could represent a first tread to develop a prognostic model to predict survival in patients undergoing surgical oncological emergencies.

In conclusion, oncological emergencies are becoming increasingly common. It is important that acute care surgeons consider a patient’s nutritional and functional status as well as cancer stage and future treatment options. Chronological age is important but not so crucial. Preoperative assessment of LDH and albumin levels could help to predict short-term outcomes when facing patients with oncological emergencies. Finally, the presence of a specific acute care surgery department has led to an awareness of these pathologies with the aim to improve the residual quality of life of the patients.

## Data availability statement

The raw data supporting the conclusions of this article will be made available by the authors, without undue reservation.

## Ethics statement

The studies involving humans were approved by Department of Surgery, Oncology and Gastroenterology (Discog). The studies were conducted in accordance with the local legislation and institutional requirements. Written informed consent for participation was not required from the participants or the participants’ legal guardians/next of kin in accordance with the national legislation and institutional requirements.

## Author contributions

AF: Conceptualization, Data curation, Formal Analysis, Project administration, Supervision, Writing – original draft. CS: Funding acquisition, Supervision, Writing – review & editing. FR: Conceptualization, Data curation, Formal Analysis, Writing – original draft. IZ: Writing – review & editing. LV: Conceptualization, Formal Analysis, Writing – review & editing. SS: Writing – review & editing. AA: Data curation, Validation, Writing – review & editing. VV: Validation, Writing – review & editing. CR: Investigation, Writing – review & editing. NB: Project administration, Visualization, Writing – review & editing. MV: Project administration, Supervision, Writing – review & editing. GD: Project administration, Supervision, Writing – review & editing.
